# Burnout syndrome assessment among Spanish oral surgery consultants: A two populations comparative pilot study

**DOI:** 10.4317/medoral.24725

**Published:** 2021-12-07

**Authors:** Carlos M Cobo-Vázquez, Celia Martín, Luis Miguel Sáez-Alcaide, Cristina Meniz-García, Laura Baca, Pedro Molinero-Mourelle, Juan López-Quiles

**Affiliations:** 1 Associate Professor, DDS, MSc, PhD. Department of Medicine and Orofacial Surgery. Faculty of Dentistry. Complutense University of Madrid, Spain; 2 Associate Dentist, Department of Dentistry and Stomatology, Gregorio Marañón University General Hospital, Madrid, Spain; 3 Assistant Professor, DDS. Department of Prosthesis. San Pablo CEU University, Madrid, Spain; 4 Assistant Professor, DDS, MSc. Department of Medicine and Orofacial Surgery. Faculty of Dentistry. Complutense University of Madrid, Spain; 5 Assistant Professor, DDS, MSc. Department of Reconstructive Dentistry and Gerodontology, School of Dental Medicine, University of Bern, Switzerland

## Abstract

**Background:**

The professional Burnout Syndrome (BOS) or Burnout is considered a professional disease made up of three interrelated dimensions (emotional exhaustion, depersonalization and lack of personal fulfillment). BOS has been documented to most severely affect the healthcare professions, especially dentists. On the other hand, its appearance has been documented at an early age, during dental training. However, there are no studies that analyze its incidence in professionals dedicated to Oral Surgery and Implantology, determining the age of onset and related factors.

**Material and Methods:**

The modified Maslach questionnaire was carried out anonymously among the professors and students of the Master of Oral Surgery and Implantology at the Complutense University of Madrid. A total of 36 participants were enrolled in this study and the results of the modified Maslach Questionnaire were established into four groups [1st year (n=6), 2nd year (n=6), 3rd year (n=6) postgraduate students and clinical teachers (n=18)]. The following variables were recorded: Age, sex, years of experience, weekly hours of work, dedication on weekends and scope of work. The statistical analysis performed included Pearson's correlation, analysis of variance, Student's t-test, F-Anova, Chi-Square and Gamma correlation. Statistical Significance of the tests was established of p≤0.05.

**Results:**

36 questionnaires were analyzed, of which 22.2% (n = 8) presented BOS, and 77.8% (n = 28) a medium risk of suffering it. The mean values and standard deviation ​​of emotional exhaustion (7.50 ± 2.43; 9.83 ± 4.12; 15.83 ± 6.21; 30.22 ± 7.86), depersonalization (5.50 ± 1.23; 50 ± 3.27; 11.33 ± 1.75; 17.56 ± 4.13), low personal fulfillment (39.67 ± 3.72; 39.33 ± 2.34; 43.17 ± 3, 55; 37.33 ± 5.51) and professional burnout (54.33 ± 2.66; 61.67 ± 2.88; 70.33 ± 5.43; 85.11 ± 9.05) in the four groups respectively. A significant association was found in the appearance of emotional exhaustion and depersonalization, years of experience, weekly work hours and the work environment.

**Conclusions:**

BOS is a disease that can appear from 30 years of age, after 5 years of professional experience and when there is a clinical consultation of 40 hours a week. Oral Surgery and Implantology seems to be a risk activity for the manifestation of depersonalization.

** Key words:**Burnout syndrome, oral surgery, dental implants, students, clinical teachers, dental education.

## Introduction

The Burnout Syndrome (BOS) was first described in 1974 by Herbert Freudenberger and was defined as a gradual erosion of the person as a consequence of chronic work stress, influenced by schedules, professional responsibility, patient demands and other environmental factors. In 1976, Maslach described the main signs and symptoms derived from stress and pressure anxiety in the care work environment and its clinical manifestations included physical, mental and somatic alterations, such as numb feeling, tired and exhausted.

From these findings, several studies have described health professions as those with the highest probability of developing BOS (1).

A report published by the Spanish National Institute of Statistics, showed that dentistry was of the professions with the highest suicide rate in the last 10 years, reported as possible consequence a combination of several etiological factors, where stress played the leading role.

Studies of United Kingdom dentists (1) also reported that stress, exhaustion and psychological distress derived from their work lead a decrease in well-being. Regarding dental clinical areas, general dentists and particularly those who are dedicated to oral surgery and implant dentistry are continually subjected to emergency, stress situations, to unsatisfied or fearful patients and a clinical activity with long hours. The stressful situations can affect clinical performance and productivity, causing mental and / or emotional exhaustion, irritation, tiredness, lack of concentration and even health problems.

The professional Burnout Syndrome is defined as a state in which the affected person experiences three interrelated dimensions described as emotional exhaustion, depersonalization and lack of personal fulfillment. Emotional exhaustion is a feeling of heaviness, exhaustion and collapse in a person's life. This tiredness prevents workers from engaging in their work on an emotional level due to their perceived lack of energy. Second, depersonalization refers to the development of negative feelings and behaviors towards other people. It often involves blaming others for one's own problems and manifests itself as cynicism, self-criticism, devaluation, self-sabotage, and disregard for the scope and value of one's work. Lack of personal fulfillment is identified as a decrease in personal self-esteem, frustration of expectations and manifestations of stress at a physiological, cognitive and behavioral level, leading to feelings of unhappiness and dissatisfaction (2).

The period of years of experience where there may be more risk or more vulnerability of appearance of BOS in dentistry is still unknown, however, a study carried out in Brazil showed the risk of students to suffer from this condition. In addition, previous studies showed that some students, from an early age, may begin to develop a tendency to suffer from BOS due to their theoretical and practical training.

Although the literature has described the multifactorial etiology of the BOS in clinicians and dental students (2), there is a lack of studies describing the period of years of experience where there may be more vulnerability of appearance or development of the condition. Therefore, the aim of the present study is to compare the Maslach questionnaire values of BOS among a sample of students and professors of an Oral Surgery and Oral Implantology university postgraduate program in order to assess the period of years of experience where there may be more risk or more vulnerability of appearance of BOS, as well as to analyze which factors have greater relation with the obtained results.

## Material and Methods

A comparative cross-sectional study based on anonymous surveys was conducted following the ethical approval (Ethical Committee N° 20/146-E) provided by the Ethical Committee of Hospital Clínico San Carlos, Madrid, Spain, and was performed in accordance with the provisions of the Declaration of Helsinki (World Medical Assembly) and an informed consent was obtained from all participants in writing prior to conducting the research.

The sample were selected within two groups, oral surgery residents (A), oral surgery teachers (B). Within this groups, to be included in the study the subjects had to fulfill the following inclusion criteria: Dentist with a Spanish dental diploma, current residents of the postgraduate program in oral surgery and oral implantology, Complutense University of Madrid and with a clinical dental experience from 1 to 5 years, current clinical teachers of the same postgraduate program, with 5 of more years of clinical and teaching experience in oral surgery and dental implants.

The collected data were confidential and was subject to the Spanish Data Protection Law and therefore a modified Maslach questionnaire, the Maslach Burnout Inventory (MBI), a questionnaire that presents a study subject with a series of statements on feelings and thoughts in regard to his/ her interest in work (3). The questionnaire is comprised of 22 items that measure the three “burnout” components: Emotional exhaustion (EE) (High:> 27 Pts; Medium: 19-26 Pts; Low: <19 Pts), Depersonalization (D) (High:> 10 Pts; Medium: 6 -9 Pts; Low: <6 Pts) and Personal fulfillment (PF) (Fulfillment:> 40 Pts; Average: 34-39 Pts; Low: <34 Pts).The subscales (EE, D, and PF) have to be kept separate and the scores on each subscale are classified according to percentile of each scale (3). Thus, based on this classification, the Maslach questionnaire allows to classify the professional Burnout Syndrome into three groups according to the score obtained (No Risk: 0-43 Pts; Trend: 44-87 Pts; Burnout Syndrome:> 88 Pts).

The entire postgraduate program was invited to participate and a maximum period of 7 days was established for participation in the study, giving the questionnaires in conventional paper format at the delivery point where the absence of the members of the research team was ensured.

A convenience sample of 36 participants were enrolled in this study, that include all the students and teachers. The results of the modified Maslach Questionnaire were established into four groups [1st year (n=6), 2nd year (n=6), 3rd year (n=6) postgraduate students and clinical teachers (n=18)] according to their academic year and professional experience. Moreover age, gender, years of work experience, number of weekly hours of clinical consultation, weekend clinical work and scope of work (private or insurance) were recorded.

Data analysis was performed using a statistical software program (SPSS V2.0; IBM Corp, Armonk, NY). To analyze the relationship between burnout syndrome scores and its categories (emotional exhaustion, depersonalization, and personal fulfilment) with the quantitative variables (age, years of experience, and hours per week), Pearson's correlation was used.

For the relationship between the burnout syndrome scores and the categorical variables recorded assessment, the Variance Analysis was performed and, in those cases, where the variable had 2 categories (Sex, work on weekends and work environment), the Student's t test was performed and finally In which the variable had more than 2 categories (Group, age, years of experience and weekly hours), the Anova contrast test was performed.

To analyze the association between the categories of burnout syndrome and the qualitative variables (group, sex, work on weekends, work environment), Tables of frequency and percentages and the Chi-square test were performed.

For the BOS analysis with the ordinal variables (group, age, years of experience and weekly hours), the Gamma correlation coefficient was used. *P* values smaller than 0.05 were considered statistically significant.

## Results

From a total of 40 submitted Maslach surveys, 36 were finally included, 36 correctly completed modified Maslach questionnaires were received within the indicated period. Of the 36 questionnaires, 18 corresponded to teachers and 18 to postgraduate students of the Master of Oral Surgery and Implantology, including the total number of postgraduate students enrolled for each course. The mean age of the teachers was 39.3 years compared to the postgraduate students of 1st year (25.0 years), 2nd year (25.8 years) and 3rd year (27.2 years) (Table 1).

The group of students of first year reported a mean experience of 1.33 years and a weekly mean dedication of 18 hours. Second year students reported 3.33 experience years and a dedication of 23.33 hours weekly. Third year students referred 3.83 experience years and a dedication of 27.5 hours weekly. Otherwise, teachers reported a mean experience of 13.61 years and clinical dedication of 43.06 hours weekly.

**Figure 1:**
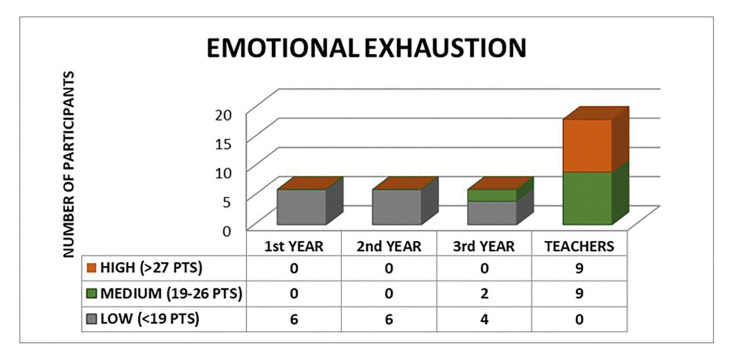
Emotional Exhaustion. Association between the categories of Emotional Exhaustion (High, Medium, Low) and the Sample Groups (Students 1st Year, 2nd Year, 3rd Year and Teachers). Chi-Square Test *p* = 0.000.

**Figure 2:**
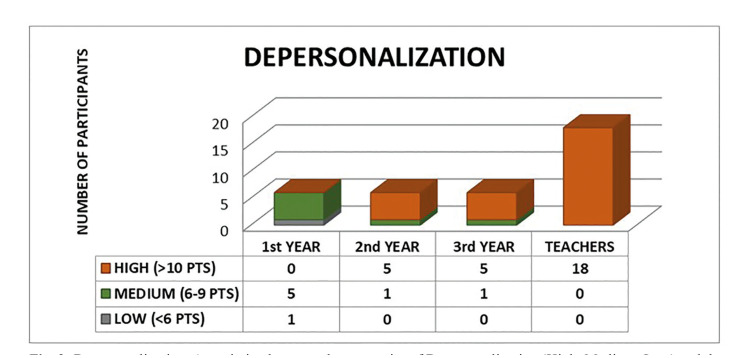
Depersonalization. Association between the categories of Depersonalization (High, Medium, Low) and the Sample Groups (Students 1st Year, 2nd Year, 3rd Year and Teachers). Chi-Square Test *p* = 0.000.

**Figure 3:**
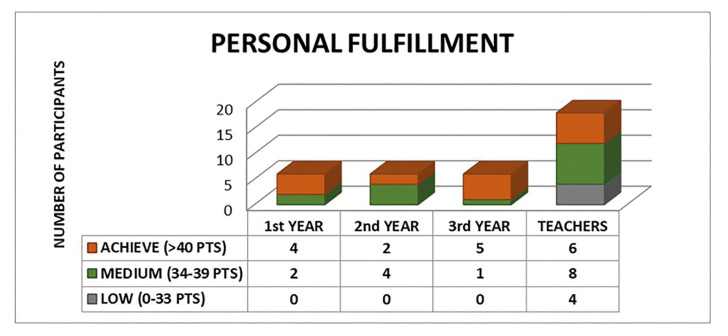
Personal Fulfillment. Association between the categories of Personal Achievement (High, Medium, Low) and the Sample Groups (Students 1st Year, 2nd Year, 3rd Year and Teachers). Chi-Square Test *p* = 0.171.

The results of analyzing the association between the burnout syndrome groups and the study groups (postgraduate students and teachers) showed an association of the sample group with emotional exhaustion and depersonalization (Fig. 1, Fig. 2 and Fig. 3).

The results of analyzing the relationship between the burnout syndrome scores and the categorical variables of two categories (Group, Sex, work on weekends and work environment) showed the existence of statistically significant differences between Postgraduate students and Teachers regarding the score on emotional exhaustion depersonalization and burnout syndrome (Table 2).

No differences were found in the burnout syndrome in terms of sex (Female: 70.26 ± 13.37; Male: 77.35 ± 14.74) and working on weekends (No: 72.47 ± 12.47; Yes: 74.43 ± 15.70).

**Table 1:**
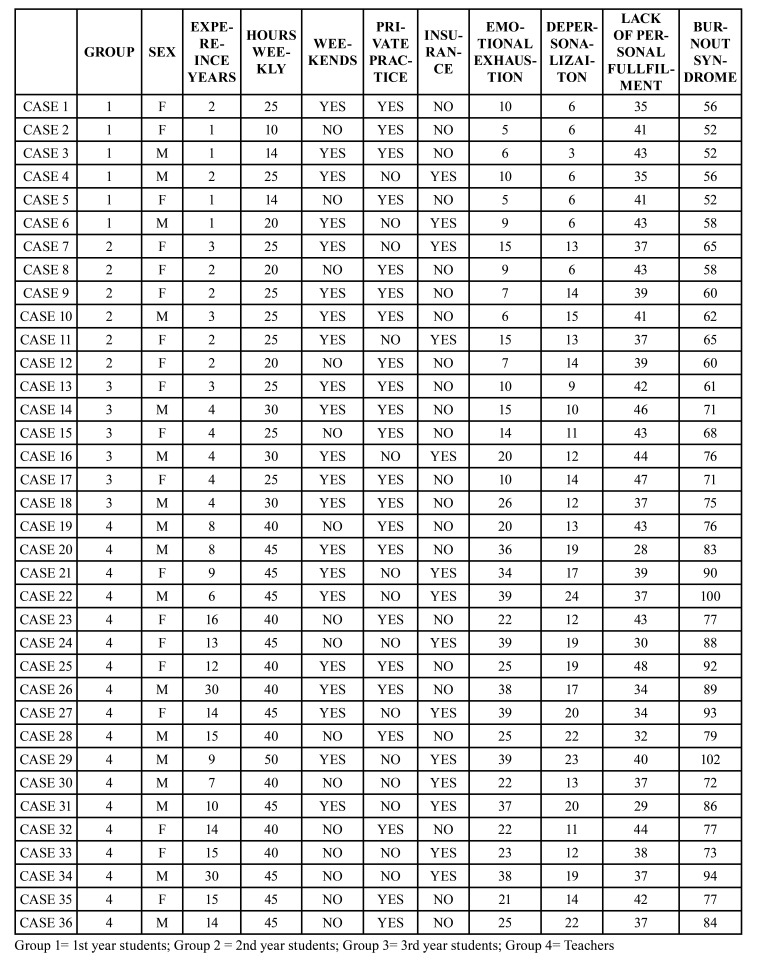
Data collected from the 36 modified Maslach questionnaires.

**Table 2:**
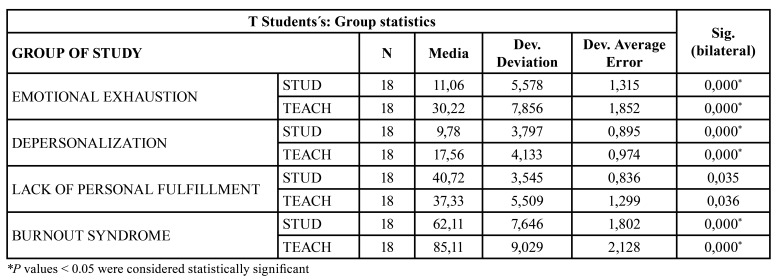
T Student test for the correlation between the scores of the variables of the Professional Burnout Syndrome and the categorical variable (Group: Student-Teacher).

On the other hand, working in the private sphere or working in a private clinic and insurance company showed significant differences in terms of emotional exhaustion, personal fulfillment, and burnout syndrome (Table 3).

A significant relationship was found between age and years of experience with emotional exhaustion and depersonalization. Weekly hours were shown to have a significant relationship with burnout syndrome (Table 4).

The analysis of the influence of the ordinal variables (age, years of experience and weekly hours) in the score of the categories of the Professional Burnout Syndrome showed the existence of statistically significant differences between the age groups, the years of experience and the hours weekly.

It can be observed that after 30 years of age (and more than 5 years of work experience) emotional exhaustion (1-5 experience years: 11.06 ± 5.58; 6-10 experience years: 32.43 ± 8.02; >10 experience years: 28.82 ± 7.80) and depersonalization (1-5 experience years: 9.78 ± 3.80; 6-10 experience years: 18.43 ± 4.39; >10 experience years: 17.00 ± 4.07) increase significantly.

The influence of weekly hours on the increase of emotional exhaustion, depersonalization and professional burnout syndrome (<20 weekly hours: 55.33 ± 3.72; 21-40 weekly hours: 71.05 ± 9.75; >40 weekly hours: 89.70 ± 7.76) can be seen from the 20 weekly hours of professional performance.

It can be seen that age groups, years of experience and weekly hours influence emotional exhaustion and depersonalization. Those professionals who work on weekends show greater emotional exhaustion. Finally, the work environment influences emotional exhaustion, being greater in those who work in private clinics and insurance companies, and in personal fulfillment, which is greater for those professionals who work in private clinics only.

**Table 3:**
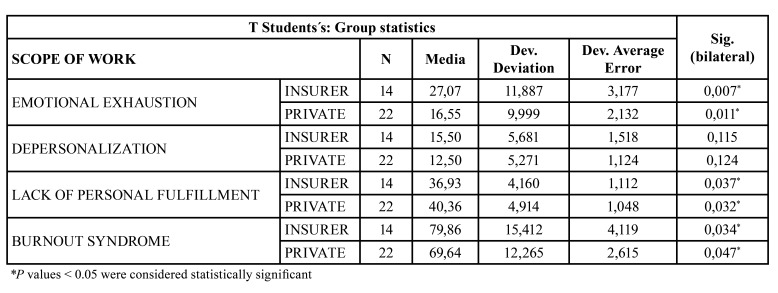
T Student test for the correlation between the scores of the variables of the Professional Burnout Syndrome and the categorical variable (Scope of work: Private Clinic- Insurer).

**Table 4:**
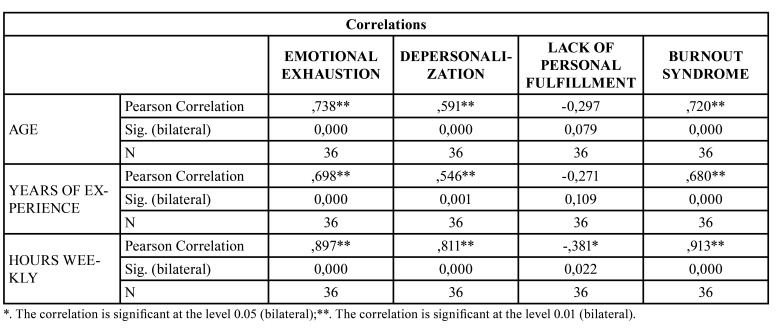
Correlation between the Professional Burnout Syndrome scores with the Quantitative variables (age, years of professional experience and weekly hours of work).

## Discussion

Burnout syndrome tends to be more common in the care or service professions, although it has been observed in all types of occupations (2).

A New Zealand study of 1,487 healthcare professionals, 32 of whom were dentists, found the highest burnout levels only behind Emergency Physicians and Psychiatrists (4). Of the 36 participants in the present study, 22.2% (n = 8) have burnout syndrome and 77.8% (n = 28) have a medium risk of suffering it. These results are somewhat lower than others, which indicate 40.5% of professionals with PDS who presented chronic symptoms (5), 42% of a study carried out in the United Kingdom with high emotional exhaustion (6,7), or the 29% reported by Huri *et al*. (8).

However, if we only attend to the group of teachers, the incidence of BOS is 50%. Regarding the aspects of the BOS, 25.0% of our sample presented emotional exhaustion, 77.8% depersonalization and 11.1% low personal fulfillment. These results are in agreement with previous studies, which show approximately that 38% of the population studied suffered emotional exhaustion, 22% depersonalization, and 12% reduced academic performance (6,9,10) or 22.1% of emotional exhaustion and 37.9% of low personal fulfillment among dentists in Brazil (11). The high percentage of professionals who showed depersonalization in our sample stands out. This fact could be due to the particularities of our population, since Oral Surgery and Implantology can be associated with a bloody act, which causes fear and stress to the patient, and this maintained over time could trigger depersonalization in dentists. As in the study of Alemany Martínez *et al*., oral surgeons seem to represent most cases of “burnout” syndrome (23). On the other hand, both the training and work of dentists have been well documented as a source of stress (6). There are studies that register 10% of postgraduate students with severe levels of "emotional exhaustion", 28% of "depersonalization" and 17% felt a "lack of personal fulfillment" (12,13).

On the other hand, burnout syndrome has been described in 17.8% of dental students according to some studies (14,15). For our part, we found that teachers present greater emotional exhaustion (30.22 ± 7.89 vs 11.06 ± 5.58), depersonalization (17.56 ± 4.13 vs 9.78 ± 3.80) and BOS (85.11 ± 9.03 vs 62.11 ± 7.65) than the postgraduate students. This increase is progressive, manifesting in such a way the values ​​of emotional exhaustion (7.50 ± 2.43; 9.83 ± 4.12; 15.83 ± 6.21; 30.22 ± 7.86), depersonalization ( 5.50 ± 1.23; 12.50 ± 3.27; 11.33 ± 1.75; 17.56 ± 4.13), low personal fulfillment (39.67 ± 3.72; 39.33 ± 2 , 34; 43.17 ± 3.55; 37.33 ± 5.51) and professional burnout (54.33 ± 2.66; 61.67 ± 2.88; 70.33 ± 5.43; 85.11 ± 9.05) in the four groups respectively.

These results are consistent with studies that have found that Burnout syndrome is more severe when dentists take their first step into the professional world (2,9,10,11). Furthermore, it has been described that dentists with a doctorate present greater emotional exhaustion than those who do not. This fact has been explained by other studies that indicate that higher academic training increases expectations and decreases satisfaction levels (12,13,16,17). In addition, according to the results presented above, the dentists who studied the specialty showed more personal fulfillment than those who already had it (8,17,18).

Our results differ from those published by Singh *et al*., Ruijter *et al*., and Jin *et al*., where they find differences between both sexes (19,20,21). According to these studies, the average emotional exhaustion among women is significantly higher than that of men; on the other hand, depersonalization in men seems to be greater than in women according to some authors. However, in our study, we did not find differences between the sexes, as did Singh *et al*., and Jin *et al*. (19,21) The probable explanation in our case is that women who engage in Oral Surgery and Implantology tend to be accustomed as much as men to stressful situations.

Regarding the factors that influence the aspects of BOS, we find that older age and more years of experience influence the increase in emotional exhaustion and depersonalization. In the present study, we found that professionals from the age of 30 and with more than 5 years of experience present high values ​​for both aspects. A previous study finds that dentists between 36-45 years suffer more emotional exhaustion and that between 46 and 55 years suffer greater depersonalization. On the other hand, they find that after 15-20 years of experience they register more emotional exhaustion and less personal fulfillment (8).

On the other hand, the greater number of weekly hours dedicated to clinical practice are manifested in this study as an increase in emotional exhaustion, depersonalization and low personal fulfillment. Like other studies (2,13,22,23), we found that all dimensions of the BOS are manifested after 40 hours of work per week. In addition, it should be noted that after 20 hours of work a week there is an increased risk of suffering emotional exhaustion, depersonalization and BOS. In a study of 314 pregraduate dental students, professional burnout was found in those students who dedicated more than 40 hours per week to their training (2). In relation to professional dedication, those dentists who dedicated their weekends to their professional activity presented greater emotional exhaustion.

Finally, in the present study differences were found in terms of the area in which the dentist carries out his activity. Those who dedicate their profession to the Private Clinic present greater personal fulfillment. On the other hand, those who work in Dental Insurance have greater emotional exhaustion and BOS. These results would be comparable with previous studies that indicate that dentists who work in the public or not very selective field face greater emotional exhaustion and depersonalization than those who work in the Private Clinic (16,20,24,25). As other studies indicate, it is common for Oral Surgeons and Implantologists who work in insurance companies to attend to more than 15 patients a day, without time for rest and in a limited physical space and an adjusted attention schedule (18,26). Furthermore, the high number of patients seen is associated with an increase in depersonalization and emotional exhaustion, according to several authors (8). It has been published that repetitive activities and with a high number of patients with a lower socioeconomic level, in a limited time of work, decreases the quality of professional practice and increases the risk of making professional errors and increases the symptoms of BOS (8,24,25,27).

The explanation for the high incidences of BOS, among others, are the care and teaching pressure they suffer in their daily tasks, the increasing demands against dentists, by dissatisfied patients, the professional practice in dental insurance clinics with a significant volume of patients and with little time to dedicate to each one of them and / or the ease of access to information through digital media, which induces erroneous knowledge in the patient.

Other authors have described as factors that caused stress among newly qualified dentists were those related to legal and insurance matters (61.2%), organization of the practice (56.6%) and personnel management (55.2%) (9,10,28). The most common stressors described in a New Zealand study were handling difficult children (52%), time pressures (48%), and maintaining high levels of concentration (43%) (29). Furthermore, stress (50%), lack of concentration and fatigue (32.9%) were the most common causes of needlestick injuries (6,30).

The present study has certain limitations. First, as it is a pilot study, the sample of subjects may not be sufficient to assume the results as an indicator of all dentists (postgraduate students and professors) who dedicate their practice to oral surgery and implantology. However, compared to other studies in university centers, the sample could be similar, taking into account the specificity of the sample. On the other hand, the personal moment when completing the modified Maslach questionnaire can influence the individual perception of the aspects that are evaluated. The possibility of individual variability must be taken into account, as well as the omission of other personal aspects that may influence the responses of the subjects, as well as the study of factors such as place of residence or remuneration for the activity.

Other important limitation is the heterogeneity of teachers sample because of age differences. In contrast to that, students have less differences about years of professional experience.

However, there are few previous studies that evaluate the period of years of experience where there may be more risk or more vulnerability of appearance of professional burnout (8,11,18,19,25). There are also no studies that critically analyze the factors of work activity that influence the appearance of burnout syndrome.

## Conclusions

Within the limitations of the present study, it is possible to conclude that BOS begins to be suffered from 30 years of age, after 5 years of professional experience and when there is a clinical consultation of 40 hours a week. This tendency is very similar in 3rd year postgraduate students with 27 hours of work per week and dedication on weekends to professional and training activities.

More studies with a larger sample may be necessary.
